# Differences in Distress Between Rural and Non-rural Appalachian Breast Cancer Patient/Caregiver Dyads During the First Year of Treatment

**DOI:** 10.13023/jah.0403.04

**Published:** 2023-01-01

**Authors:** Jordan Tasman, Callie D. McAdams, Jillian Lloyd, Ashton J. Brooks, Patricia Nola Eugene Roberson

**Affiliations:** University of Tennessee, Knoxville, jtasman@vols.utk.edu; CMcAdams@utmck.edu; JLLoyd@utmck.edu; AJBrooks@utmck.edu; University of Tennessee - Knoxville

**Keywords:** Appalachia, breast cancer, anxiety, depression, caregiver, rural, strain, treatment

## Abstract

**Introduction:**

Breast cancer patients and their caregivers living in rural Appalachia face substantial health disparities compared to their non-rural Appalachian counterparts. However, there is limited research on how these specific health disparities in rural Appalachian communities may impact patient psychological distress and caregiver strain during the first year of breast cancer treatment.

**Purpose:**

The purpose of the current study was to assess differences in patient psychological distress (depression and anxiety) and caregiver strain between rural non-rural Appalachian breast-cancer-affected dyads (patients and their caregivers) during the first year of treatment.

**Methods:**

A total of 48 Appalachian breast cancer patients (with a Stage I through Stage III diagnosis) and their identified caregiver (together, ‘dyads’) were identified from The University of Tennessee Medical Center across 2019 to 2020. Dyads completed follow-up surveys throughout the first year of treatment. In this prospective pilot study, measures on anxiety, depression and caregiver strain were self-reported and then analyzed using RM-ANOVA.

**Results:**

There was a statistically significant higher number of reports of patient depression and caregiver strain in rural-residing dyads compared to non-rural-residing dyads. However, there was not a statistically significant difference between rural and non-rural Appalachian dyads for patient-reported anxiety during the first year of treatment.

**Implications:**

The higher reported patient depression and caregiver strain among rural-residing Appalachian patients may indicate the need for implementing remote (e.g., telehealth) Cognitive Behavioral Therapy (CBT) to address the psychological needs of rural-residing dyads. Additionally, greater education from physicians to rural dyads on what to expect during treatment could alleviate caregiver strain.

## INTRODUCTION

Breast cancer patients and their caregivers (dyads) residing in rural Appalachia face a range of health disparities compared to urban counterparts. They live longer distances from healthcare resources^1^, receive different surgical recommendations^2^,^3^ and have poorer psychological outcomes.^1^ These health disparities may be due to the lower socioeconomic status and geographic isolation that characterizes the Appalachian Region.^4^,^5^ Breast cancer is the second-leading cause of cancer death among women in the U.S.; and in rural areas, like swaths of Appalachia, the mortality rate of breast cancer is higher compared to urban areas.^6^,^7^ This mortality disparity is likely due to the later stage of diagnosis^7^, limited access to specialized healthcare services and resources^2^,^3^,^6^,^8^, and lower rates of screening mammography.^9^ Rural Appalachia is identified by the National Cancer Institute (NCI) as a specialized population of interest due to its isolated geography and challenging economic conditions^5^,^9^; given this, patients and caregivers in rural areas would be expected to experience increased psychological distress and caregiver strain compared to those in urban Appalachian communities.

### Rural Breast Cancer Patients’ Psychological Distress

Breast cancer treatment contributes to mental health distress which can be more pronounced for rural patients.^10^ However, the psychological experiences among rural breast cancer patients have been studied with varying results. Surprisingly, there has been a lack of consensus on the impact of rurality and mental health outcomes on these experiences. In a study analyzing depression among breast cancer patients over a course of 13 months, Schlegel et al. did not demonstrate rurality as an independent predictor of depressive symptoms.^11^ In contrast, Burris found that rural cancer patients demonstrated poorer mental health measurements, even after controlling for differences in education and level of physicality.^12^ Additionally, Alagizy et al. found that 81% of rural breast cancer patients report anxiety symptoms compared to 19% of urban patients and that a majority of patients in rural areas experience depression compared to their urban counterparts—77.3% v. 22.9%, respectively.^10^ There are several possible reasons for these differences across studies. The definition of rurality varies across studies, which could lead to conflicting results. Additionally, some studies evaluated the psychology of breast cancer patients within the context of multiple cancers, including colorectal, prostate and lymphoma.^12^

### Caregiver Strain

Breast cancer patients rarely experience their disease in isolation. Both formal and informal caregivers can have experiences associated with the care of a cancer patient that can affect their own psychological distress. Caregiver strain is the perceived distress and decreased well-being from the responsibilities of the caregiving role.^13^ Caregivers are often required to do more than simply provide home care for the patient; frequently they are the primary resource for traveling to appointments and navigating the financial aspects of cancer treatment. This can contribute to increased rates of anxiety, depression, financial strain, and social isolation.^14^,^15^ The amount of time spent, and family adjustment required, to care for patients can cause adverse health effects and exacerbated stress among caregivers.^16^ It is not known, however, if caregiver strain is different for rural and non-rural caregivers of breast cancer patients. However, given the added barriers to patient treatment in these contexts (longer travel to healthcare facilities, higher-stage diagnoses), it is reasonable to assume that strain is increased for rural caregivers.

The existing literature provides evidence for increased barriers to accessing breast cancer treatment, higher levels of patients’ psychological distress (i.e., anxiety and depression), and caregiver strain among rural-residing dyads compared to their urban counterparts. There is limited research on how breast cancer treatment impacts dyads’ psychological distress, specifically in rural and urban Appalachian communities and on how differences in rurality may change during treatment. The present study aims to close a gap in understanding rural mental health disparities by identifying and analyzing the difference among a specific population’s—rural and urban Appalachian dyads’—psychological distress and caregiver strain during the first year of treatment. We hypothesize that (1) breast cancer patients residing in rural Appalachian counties have increased psychological distress (anxiety and depression) compared to patients in urban areas during the first year of treatment; and (2) Caregivers of breast cancer patients residing in rural Appalachian counties experience greater strain compared to their urban counterparts during the first year of treatment.

## METHODS

### Procedures

This is a quantitative prospective dyadic pilot study conducted from 2019 to 2020 on 54 breast cancer patients and their identified caregivers residing in East Tennessee’s Central Appalachian Region throughout the first year of treatment. A telephone or online survey of the recruited breast cancer dyad (patient and caregiver) was conducted based on their preferred method of contact at four different timepoints (baseline, 6 weeks, 6 months, and 12 months). Breast cancer patients and their identified caregiver (spouse, family member, friend, or other) were recruited from the University of Tennessee Medical Center Cancer Institute by a team member at their first consultation appointment with the patients’ surgical oncologist. The surgical oncologist notified the primary investigator (PI) of the timing of appointments for patients who fit the study’s inclusion criteria. The PI spoke with dyads after their consultation to inform them that they qualify for the current research study, to provide an overview of the study, and to obtain informed consent for interested dyads. Those who were not interested were thanked for their time.

Recruited patients had been diagnosed with Stage I–III estrogen-receptor positive (ER+) breast cancer; stages 0 and IV were excluded. Patients underwent either a partial mastectomy or total mastectomy as their surgical treatment. Prior to surgical treatment, a small portion of patients received neoadjuvant chemotherapy. Some patients went on to receive adjuvant radiation and/or chemotherapy based on current standards of care. All patients were prescribed anti-estrogen therapy (AET) within 6 weeks of completion of their primary treatment (surgery, radiation, or chemotherapy).

Each dyad completed their baseline survey before the patient’s primary treatment started. Subsequent surveys were completed by the dyads at 6 weeks, 6 months, and 12 months post-operatively. Response rates for breast cancer patients (n=174 total responses) was 85.71% at the 6-week mark, 79.60% at 6 months, and 89.80% at the 12-month follow-up survey. The response rate for caregivers (n= 168 total responses) was 85.42% at 6 weeks, 83.33% at 6 months, and 81.25% at the 12-month follow-up survey. All surveys for the dyads were completed individually. There was no incentive for participating in the research. The study was approved by the University of Tennessee Medical Center Institutional Review Board (IRB).

### Measures

The variables used to measure the hypotheses included patient anxiety, patient depression, and caregiver strain at each timepoint. Patient anxiety was measured using the PROMIS Item Bank v.1.0– Global Health– Emotional Distress– Anxiety– Short Form 4a^17^, which demonstrated excellent internal consistency (baseline = 0.945; 6-week = 0.904; 6-month = 0.931; 12-month = 0.911). Patient depression was measured using PROMIS Item Bank v1.0– Emotional Distress– Depression– Short Form 4a^17^. PROMIS Depression additionally represented excellent internal consistency for patient depression (baseline = 0.852; 6-week = 0.940; 6-month = 0.945; 12-month = 0.941). PROMIS Anxiety and PROMIS Depression are both 4-item 5-point Likert scales with responses ranging from “never” (1) to “always” (5). Caregiver strain was measured using the Caregiver Strain Index developed by BC Robinson (1983) with a higher score indicating a greater level of strain.^18^ The CSI demonstrated strong internal consistency (baseline = 0.861; 6-week = 0.801; 6-month = 0.883; 12-month = 0.896). The caregiver strain index is a 12-item nominal scale with response options consisting of “yes” (1) or “no” (2). The variable for rurality was collapsed into a dichotomous variable (1 = rural, 2 = suburban/urban) from the survey options (1 = rural, 2 = suburban, 3 = urban).

### Data Analytic Strategy

The data collected from all four time points were analyzed using IBM SPSS 27.0 software. In SPSS, a mean replacement for patient anxiety, patient depression, and caregiver strain was conducted with reported patient rurality to account for missing data. A full 86.15% of the original missing data was replaced for patient anxiety, 86.15% for patient depression, and 86.36% for caregiver strain. After completion of the mean replacement to handle the missing data (n=31)^19^, three repeated measures analyses of variance (RM-ANOVA) were conducted to test the hypotheses for each of the variables of interest (patient depression, patient anxiety, and caregiver strain). Intra-cluster correlation was automatically calculated in the RM-ANOVA, and no adjustments were made to the degrees of freedom, given that the assumptions were all met. Additionally, the standard error utilized for all three outcome variables was the Partial Eta Squared in the RM-ANOVA with small effect equal to 0.01, medium effect equal to 0.06 and large effect greater than or equal to 0.14.^20^ Rurality was included as a covariate to test differences in change in the variable of interest. Huynh-Feldt correction was utilized to assess significance for all three outcomes, since Mauchly’s Sphericity Test was violated with *p* < 0.05 and Epsilon greater than 0.750. A post-hoc analysis was conducted to assess the significance at each time point.

## RESULTS

The average age for patients was 63 years (range: 32–83 years). Patient sex was identified as 2% male (n=1) and 98% female (n= 53). Patient race was predominately white (91%), with 2% of patients identifying their race as black, 2% as Asian American, and 2% of as “other.” Caregiver’s self-reported race consisted of 98% white, and the remaining 2% of caregivers self-reported their race as “other.” Caregivers’ reported relationship to patients in the study consisted of spouse (63%), family member (24%), friend (9%), and other types of relationship (4%). Patients’ self-reported rurality consisted of 57.1% residing in urban/suburban areas and 43% in rural locales. Oncological characteristics included patients at stage I (54%), stage II (33%), and stage III (11%). The proportion of patients that underwent a partial mastectomy v. total mastectomy was 69% and 31%, respectively. Additionally, 63% of patients received adjuvant radiation, and 15% underwent neoadjuvant chemotherapy, while 24% received it in the adjuvant setting.

The assumption of the equality of variances and normality was met for all outcome variables at all timepoints, except for baseline (pre-treatment) caregiver strain. Post-hoc analyses examine the non-parametric test for this time point.

**Figure 1:**
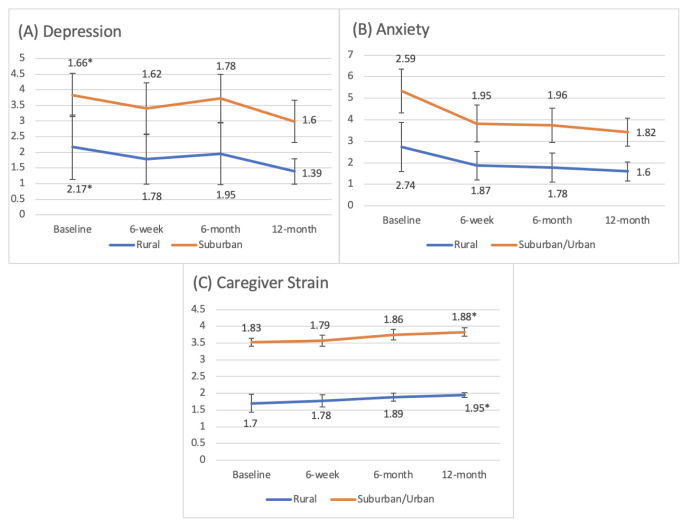
Post-hoc analyses for the interaction among Patient Depression, Patient Anxiety and Caregiver Strain across all four timepoints. NOTE: *p < 0.05.

Interaction effect analysis demonstrated that patients who reported living in rural communities had significantly higher depression compared to their urban counterparts throughout the first year of treatment (*F*(2.790, 136.729) = 3.888, *p* = 0.012). The interaction effect for caregivers of patients who reported living in rural communities had significantly higher strain throughout the first year of treatment compared to their urban counterparts (*F*(2.567, 102.694) = 5.41, *p* = 0.003). The trajectory of caregiver strain throughout the first year of treatment demonstrated a continuous gradual increase for caregivers of patients in rural communities compared to those of urban communities who demonstrated an initial decrease and then slight increase in caregiver strain (Fig. 1).

The interaction effect between patient-reported rurality and patient anxiety demonstrated no statistically significant difference (*F*(2.750, 134.728) = 0.866, *p* = 0.453). The trajectory of anxiety was very similar for rural and urban patients, with high anxiety levels at baseline followed by a sharp decrease. RM-ANOVA results for all three outcomes are demonstrated in Table 1.

### Post-hoc Analyses

A series of t-tests were conducted for post-hoc analyses in SPSS to examine non-parametric statistical differences (*p* < 0.05) of rural status at each of the time points for hypothesis 1 and hypothesis 2.

For hypothesis 1, depression was significantly higher (*t*(49) = 2.105, *p* = 0.040) among rural breast cancer patients (M = 2.1673, SD = 1.6600) compared to their suburban/urban counterparts (M = 1.66, SD = 0.69) at baseline only. Although the results were not significant at the additional timepoints, rural patients reported higher depression at 6 weeks and 6 months, as well. Anxiety was not statistically significant at any time point for patients in rural v. suburban/urban communities.

For hypothesis 2, caregiver strain was significantly higher (*t*(40)= 2.262, *p* = 0.03) among caregivers of rural patients (M = 1.95, SD = 0.06) compared to caregivers of non-rural patients (M = 1.88, SD = 0.12) at the 12-month time point only. All other timepoints for hypothesis 2 were not statistically significant; however, at the 6-month time point, rural caregiver strain was not significantly higher than urban caregiver strain. The results from the t-test post-hoc analyses at each timepoint for patient depression, patient anxiety and caregiver strain are shown in Table 2.

## IMPLICATIONS

The present study is among the first to compare patient psychological distress (anxiety and depression) and caregiver strain between rural and urban Appalachian dyads across the first year of treatment. There is a gap in the current literature examining psychological distress among specifically oncological patients utilizing the PROMIS Depression and Anxiety scales. Overall, the descriptive data in the current study can be compared to the general population using PROMIS Depression and Anxiety. According to Pilkonis, the initial basis for their study suggests that a skewed mean score for Depression and Anxiety is 1.23 and 1.28, respectively.^17^ That the current study having higher mean scores at 2.167 and 1.95 demonstrates the difference in psychological distress between oncologic patients and the general population. The caregiver strain index has been analyzed in caregivers of patients with advanced cancer, and this study demonstrates that the mean score is 4.2.^21^ This is considerably higher than the mean scores for both rural and suburban/urban caregivers’ CSI mean in the current study: 1.95 and 1.88, respectively. This largely in part is due to the study's use of advanced cancer patients compared to our population of Stage I–III ER+ breast cancer. Further research should expand on the current scales to compare rural and suburban/urban differences among patients and caregivers.

Results show that rural-residing patients have higher levels of depression, but only statistically significant at baseline, which was before treatment. Despite the non-statistical significance at the remaining time points throughout the first year of treatment, rural-residing patients’ reports of depression do trend higher than their non-rural counterparts, except for at the 12-month follow-up. Interestingly, at 12 months, there is evidence that non-rural patients may experience more depressive symptoms. Nevertheless, it may be beneficial for breast oncologists to offer behavioral health interventions through remote delivery options (i.e., telehealth) for rural patients who live further away from their office; these options may also benefit non-rural patients. Breast oncologists may consider cognitive behavioral therapy (CBT) interventions that can improve depressive symptoms during treatment.^22^ CBT has effective results in reducing depression in breast cancer patients during treatment.^22^,^23^ The implementation of CBT interventions through telehealth may be critical for this rural Appalachian population, who can experience geographic isolation from healthcare resources. Additionally, partnering with community resources—such as local churches, schools, and local public health centers—to implement CBT could also be an effective way to reach rural Appalachian patients and family members.

Contrary to our expectation, the main effect for anxiety is not statistically significant among rural and non-rural patients throughout the first year of treatment. These findings are inconsistent with the results of Alagizy et al., in which rural breast cancer patients have higher levels of anxiety than urban ones.^10^ These results may differ because the present study’s self-reported measure of rurality may be inconsistent with the patients’ actual rurality statuses.

It appears that, for rural caregivers, strain continuously increases throughout the first year of treatment; their non-rural counterparts experience strain, but at a consistent rate. The gradual increase in caregiver strain for rural caregivers may be attributed to a lack of adequate preparation from physicians on what to expect during treatment, given the burden of travel times and worse access to healthcare facilities. Giving additional education to rural patients and family members on the challenges of breast cancer treatment may help to alleviate caregiver strain. Further research is necessary to target what specific aspects of caregiver strain (financial, physical, psychological, social, or personal) are more prevalent and distressing for rural caregivers.^13^ Several studies demonstrate that simple educational and emotional support programs can greatly improve the caregiver experience.^24^,^25^,^26^ This would allow for implementation of specific resources in rural Appalachian communities to alleviate strain in caregivers during breast cancer treatment.

With the impact of breast cancer treatment on both patients and caregivers, consideration for systemic cognitive behavioral or psychosocial interventions to target the dyad simultaneously may be an effective way to improve psychological distress and alleviate caregiver strain.^23^ Overall, these findings demonstrate the influence that place of geographical residence (rural v. urban) has on mental health outcomes and the need to improve access to cognitive behavioral health interventions for breast cancer patients and caregivers during treatment.

### Limitations

While the current study offers critical implications on the differential impact of breast cancer treatment on patient’s psychological distress and caregiver strain among patients and caregivers in rural v. urban Appalachia, it has several limitations. A primary limitation is that patient rurality (rural v. urban/suburban) was collected based on self-reported data from the baseline survey, which may lead to inaccurate reports of geographical residence. Future research examining the influence of rurality on mental or physical health outcomes should strongly consider utilizing RURA, RUCC, UIC or census tracking for a more accurate assessment of rurality. Second, the current sample was small, so future research should replicate this data on a larger sample size. Additionally, patients’ psychological distress and caregiver strain were self-reported measures, which could be biased. The study protocol utilized the same survey measurements to help limit self-reported bias. Lastly, while both telephone and online options were provided to ease participant burden, this also may have introduced bias, as the dynamic is different with researcher-guided questioning and interaction. Unfortunately, data was not collected on whether participants took park via phone vs. survey, but estimates are that participation via phone was minimal (approximately less than 10%).

**SUMMARY BOX**

**What is already known about this topic?**

Breast cancer patients and caregivers residing in rural areas experience exacerbated health disparities, including longer distances to healthcare resources and poorer psychological distress (anxiety and depression).

**What is added by this report?**

There is limited research comparing psychological distress and caregiver strain, specifically among rural and urban Appalachian breast cancer patients and caregivers. The current study demonstrates that rural Appalachia-residing patients and caregivers have higher levels of depression and strain.

**What are the implications for future research?**

Rurality can negatively impact breast cancer patients’ psychological distress and caregiver strain throughout the first year of treatment. Interventions designed to simultaneously target rural-residing dyads could help alleviate distress during treatment.

## Figures and Tables

**Table 1:** Demographic characteristics of rural and suburban/urban patients and caregivers (N=108)

	Patient N=54		Caregiver N=54	
	*Rural* N=18 (%)	*Suburban/Urban* N=24 (%)	*Rural* N=18 (%)	*Suburban/Urban* N=24 (%)
**Race**				
White American	88.9%	91.7%	77.8%	66.7%
Black American	5.6%	4.2%	0%	0%
Asian American	0%	4.2%	0%	0%
Other	5.6%	0%	0%	0%
**Education**				
Less than high school	0%	0%	0%	4.2%
Highschool/GED	22.2%	16.7%	5.6%	8.3%
Some college	16.7%	54.2%	16.7%	16.7%
4-year degree	33.3%	16.7%	27.8%	25.0%
MS or PhD	27.8%	12.5%	27.8%	12.5%
**Employment**				
Full-time	33.3%	29.2%	27.8%	37.5%
Part-time	0%	8.3%	5.6%	4.2%
Home maker	0%	12.5%	11.1%	25.0%
Retired	55.6%	41.7%	16.7%	66.7%
Other	11.1%	8.3%	16.7%	33.3%
**Household Income**				
Less than $10,000	0%	0%	0%	0%
$10,000–$19,000	0%	0%	0%	0%
$20,000–$29,000	0%	0%	0%	0%
$30,000–$39,000	0%	4.2%	0%	0%
$40,000–$49,000	11.1%	0%	5.6%	0%
$50,000–$59,000	11.1%	4.2%	0%	4.2%
$60,000–$69,000	11.1%	8.3%	11.1%	8.3%
$70,000–$79,000	5.6%	8.3%	11.1%	4.2%
More than $80,000	55.6%	66.7%	50.0%	41.7%

**Table 2:** Repeated measures – Analysis of Variance (RM-ANOVA) Results for the interaction effect among Patient Depression, Patient Anxiety, and Caregiver Strain with Patient Rurality (N= 108)

	Baseline M (SD)	6-week M (SD)	6- month M (SD)	12- month M (SD)	F-stat.	*P-*value	df, error†	Effect Size§	Power¶
**Patient**					3.888	**0.012** *	2.790, 136.73	0.074	0.769†
**Depression**									
Rural	2.17 (1.03)	1.78 (0.80)	1.95 (0.98)	1.36 (0.40)					
Suburban/Urban	1.66 (0.69)	1.62 (0.83)	1.78 (0.77)	1.60 (0.69)					
**Patient Anxiety**					0.866	0.453	2.750, 134.73	0.017	0.226†
Rural	2.74 (1.14)	1.87 (0.66)	1.78 (0.69)	1.60 (0.44)					
Suburban/Urban	2.59 (1.01)	1.95 (0.86)	1.96 (0.80)	1.82 (0.65)					
**Caregiver Strain**					5.411	**0.003** *	2.567, 102.69	0.119	0.897†
Rural	1.70 (0.27)	1.78 (0.18)	1.89 (0.12)	1.95 (0.07)					
Suburban/Urban	1.83 (0.12)	1.79 (0.17)	1.86 (0.16)	1.88 (0.12)					

NOTES: Post-hoc difference in means are depicted in Fig. 1.

* *p* < 0.05

† df is assessed using non-parametric tests: Huynh-Felt correction was used to assess df, since Mauchly’s Sphericity Test was violated.

§ Effet size is assessed with η^2^: small effect η^2^ = 0.01; medium effect η^2^ = 0.06; large effect η^2^ = 0.14

¶ Observed Power
